# Essential Oils from *Humulus Lupulus* scCO_2_ Extract by Hydrodistillation and Microwave-Assisted Hydrodistillation

**DOI:** 10.3390/molecules23112866

**Published:** 2018-11-03

**Authors:** Katarzyna Tyśkiewicz, Roman Gieysztor, Marcin Konkol, Jan Szałas, Edward Rój

**Affiliations:** 1 Supercritical Extraction Department, New Chemical Syntheses Institute, Al. Tysiąclecia Państwa Polskiego 13A, 24-110 Puławy, Poland; roman.gieysztor@gmail.com (R.G.); marcin.konkol@ins.pulawy.pl (M.K.); edward.roj@ins.pulawy.pl (E.R.); 2 Import Export J.A. Szałas Company, Garbarska 125A, 26-600 Radom, Poland; jan.szalas@chmiel.com.pl

**Keywords:** essential oil, extraction techniques, hops extracts, hydrodistillation, Marynka strain, microwave-assisted hydrodistillation

## Abstract

Two different extraction methods were used for a comparative study of essential oils obtained from the *Humulus lupulus* scCO_2_ (sc-supercritical) extract: microwave-assisted hydrodistillation (MAHD) and conventional hydrodistillation (HD). As a result, the best conditions for the maximum essential oil production were determined for the MAHD method at 335 W microwave power for 30 min at water to raw material ratio of 8:3. The obtained essential oils were enriched in β-myrcene in the amount of 74.13%–89.32% (wt%). Moreover, the first application for determination of the above-mentioned volatile compounds by supercritical fluid chromatography (SFC) with photo-diode array detection (PDA) is presented, which in comparison with gas chromatography coupled with mass spectrometry (GC-MS/MS) resulted in similar values for β-myrcene and α-humulene in obtained samples within less than 1 min.

## 1. Introduction

Hops (*Humulus lupulus*) belong to a perennial plant, which is mainly used in the brewing industry due to its valuable properties [1,2]. These properties are attributed to alpha- and beta-acids (responsible for bitterness), essential oils, and polyphenols, which constitute 24, 1, and 5 wt%, respectively [3,4]. The above-mentioned groups of compounds are considered as secondary metabolites. The phenolic compounds present in hops have been investigated in terms of their anticarcinogenic, antioxidant, and anti-inflammatory properties [5,6]. Secondary metabolites’ composition affects the assessment of the diversity of a given species. However, according to Biendl et al. [3] the exact functions of these metabolites are yet unknown. It is believed that they protect the plant against pests and pathogens. Due to their favorable physiological and pharmacological properties, secondary metabolites are used in the pharmaceutical industry as medicines and dietary supplements. The antibacterial properties were also evaluated for hops extract obtained by the supercritical fluid extraction [7]. 

The main methods used to obtain essential oils are, among other, hydrodistillation, steam distillation, maceration, absorption, and supercritical extraction. These methods are referred to as conventional methods [8]. In order to shorten the extraction time and improve the extraction efficiency, another form of hydrodistillation has gained a lot of interest. Microwave-assisted hydrodistillation (MAHD), which is an advanced distillation method using microwave heating, has an advantage over conventional hydrodistillation as was indicated by a number of researchers [8,9,10,11]. It is a technique widely used to extract essential oils from medicinal plants and herbs due to its economic and environmental advantages (‘green’ technology) [12]. It is used in the extraction of various chemical compounds from the group of aromatic compounds, phenolic compounds, pesticides and, especially, essential oils. It is proven that the 30-minute duration of the microwave hydrodistillation process corresponds to a duration of conventional hydrodistillation that is eight times longer [9]. Hydrodistillation and microwave-assisted hydrodistillation have been compared in terms of extraction time, extraction efficiency, chemical composition, and quality of the essential oil as well as energy consumption. Microwave action is based mainly on the weakening of cell membranes and facilitating the extraction of biologically active compounds [13]. 

Literature data confirm the superiority of the microwave assisted hydrodistillation over conventional hydrodistillation. In previous studies, microwave-assisted hydrodistillation was carried out on such materials as ginger [12], lemongrass [12], rosemary [14,15], cinnamon [8,16], mint [10], thyme [9], sandalwood [11], and orange [17]. However, there is no information available on the use of microwave hydrodistillation in order to obtain essential oils from hop extracts, and also to compare this method with hydrodistillation with the example of the mentioned materials. The mixture of hop extract and water required the addition of residues as a scattering element obtained after the scCO_2_ extraction of *Humulus lupulus*. 

**Figure 1:**
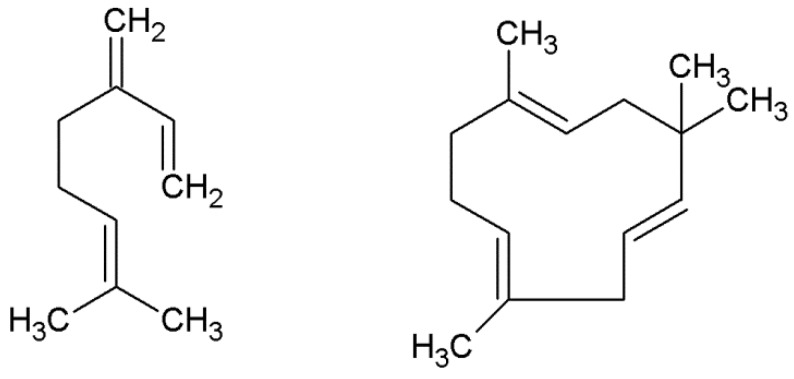
The chemical structures of β-myrcene and α-humulene.

β-myrcene and α-humulene, which belong to a group of terpenes, are characterized by their high oxidative sensitivity. During the brewing processes, thermal oxidation products of β-myrcene and α-humulene may affect both the quality of beer and its organoleptic properties [18]. Therefore, their use as beer additives is limited. However, if not in brewing, the oil rich in β-myrcene and α-humulene might be used in medicine [19]. For instance, β-myrcene is responsible for the anti-inflammatory effects in human osteoarthritis treatment [20]. The activity of α-humulene is described as similar to that of dexamethasone (anti-inflammatory, antiallergic, immunosuppressive) [21]. Figure 1 presents the chemical structures of β-myrcene and α-humulene. 

## 2. Materials and Methods

### 2.1. Plant Material

*Humulus lupulus* scCO_2_ extracts were used as experimental raw materials in this study. The extracts were obtained with the use of supercritical fluid extraction with carbon dioxide in a supercritical state from the Polish hops variety, Marynka. Hop cones from the 2017 harvest were used to produce the hop extract, “Marynka”. The geographic origin of crops was the Lublin province, Poland. Cones with a moisture content of 10.6% were milled on a Retsch SM100 mill using a sieve with a mesh size of 1.5 mm. The average particle size was 0.8 mm. The produced material was subjected to the extraction with supercritical carbon dioxide using a pilot plant. A total of 4 kg of ground hop cones were loaded into a 20 dm^3^ extractor. The extraction was carried out with the following parameters: Pressure −250 bar, extraction temperature −50 °C, and CO_2_ consumption −50 kg/kg dm. The extraction yield was 11.4% by weight.

### 2.2. Sample Preparation

The samples for microwave-assisted hydrodistillation (MAHD) and hydrodistillation (HD) were prepared in the following way. The extract (ca. 150 g) was weighed separately in a 1 L flask and approx. 400 g of distilled water was added and the resulting emulsion was stirred for half an hour. Water to extract ratio was 8:3. For the experiments, 50 g of residues (residues obtained after the extraction of hops with scCO_2_) were also added to the mixture. Such preparation of the material assures an optimal increase of the material contact with the solvent and enables satisfactory efficiency. Table 1 summarizes the exact compositions of the mixtures for microwave-assisted extraction and hydrodistillation for Marynka extract. For the sake of clarity and discussion, the oils were named according to the scheme: the name of the extract + power at which the extraction was carried out or the name of hydrodistillation apparatus (e.g., Marynka_275 W).

### 2.3. Microwave-Assisted Hydrodistillation (MAHD)

The MAHD extractions of the essential oils from Marynka hops varieties were performed at atmospheric pressure using a NEOS-GR (Milestone Technologies) system, equipped with a glycol cooled condenser, lab-grade glycol chiller, collected specimens, as well as a video system for the visual control of the process. The extraction was continued at 100 °C until no more essential oil was obtained. The essential oils were dried over anhydrous sodium sulfate, and filtered and stored in dark vials at 4 °C. During the process, two parameters were tested, namely, microwave power as well as the influence of the extraction time (HD and MAHD) on the oils composition and obtained yields. 

The efficiency of the processes was calculated from the amount of the extracts loaded into the extraction process (*m_e_*) to the amount of obtained essential oils (*m_oil_*) [4]. The yields (*Y*) of extractions were calculated according to the following equation (Equation (1)):

(1) $$\;Y\;\left(\%\right)=\;\frac{m_{e}}{m_{oil}}\times 100\%\;$$

The total energy used for the extraction of essential oils by means of MAHD method was calculated according to the following equation (Equation (2)):

(2) $$\;EC=\;\frac{P\;\times t}{1000}\;$$

in which, *EC* (kWh) is the energy consumed, *P* (W) is the power consumed during the extraction, and *t* (h) is the extraction time. 

### 2.4. Hydrodistillation (HD)

The mixture was subjected to hydrodistillation at 150 °C until no more essential oils were obtained, which was approx. 276 min for Marynka using a Deryng apparatus. The heating was performed using a heater equipped with a magnetic stirrer (Heidolph, Schwabach, Germany) with 825 W and working temperature up to 300 °C. The extracted essential oils were dried over anhydrous sodium sulfate, and filtered, weighed and stored in dark sealed vials at 4 °C. 

## 3. Analytical Procedure

### 3.1. Supercritical Fluid Chromatography (SFC)

**Figure 2:**
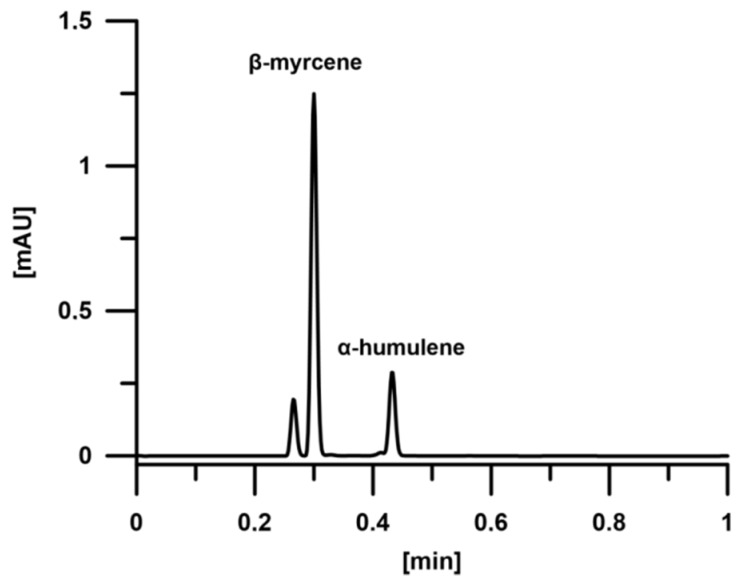
The chromatogram of β-myrcene and α-humulene by UPC^2^ (ultra-performance convergence chromatography) on HSS C18 SB (100 mm × 3.0 mm; 1.8 μm) column; 35 °C, ABPR (automated back pressure regulator): 12.6 MPa; pure CO_2_; isocratic elution.

The separation of β-myrcene and α-humulene was conducted on a Waters Acquity Ultra-Performance Convergence Chromatography (UPC^2^) system (Waters, Milford, MA, USA) equipped with a PDA detector. For the data analysis, Empower 3 software was used. The column was Acquity UPC^2^ HSS C18 SB (100 mm × 3.0 mm; 1.8 μm), packed with a chemically modified, highly strengthened silica with C18 groups. Carbon dioxide (99.998% purity), which was used as the mobile phase in SFC, was purchased from Air Liquide. The separation was performed with an isocratic elution with pure CO_2_ and a flow rate of 2.3 mL/min as well as a column temperature of 35 °C and ABPR (automated back pressure regulator) set to 12.6 MPa. The compensation wavelength was set at 500−600 nm throughout the analyses. The wavelength for the compounds was set to 220 nm. The authentic analytical standards of β-myrcene and α-humulene were purchased from Sigma-Aldrich and were dissolved in hexane. Figure 2 presents the chromatogram of β-myrcene and α-humulene by UPC^2^ method.

#### 3.1.1. Quantification

In order to quantify β-myrcene and α-humulene in hops oils, an external calibration method was applied. Individual stock solutions of authentic standards of β-myrcene and α-humulene were prepared in hexane. The appropriate amounts of β-myrcene and α-humulene were weighed and hexane was increased to the volume of 1 mL. The standard curves were obtained from five concentrations in the amount of 1000, 500, 250, 125, and 62.5 μg for β-myrcene and 500, 250, 125, 62.5, and 31.125 μg for α-humulene, adjusted to the real content of β-myrcene and α-humulene in the analyzed samples. 

#### 3.1.2. Partial Method Validation

Limits of detection (LOD), limits of quantification (LOQ), as well as repeatability and intermediate precision, were determined for the mixture of β-myrcene and α-humulene in order to evaluate the correctness of the provided method. Limits of detection (LOD) were 0.78 μg/mL for β-myrcene and 0.89 μg/mL for α-humulene, whereas limits of quantification were, respectively, 2.23 μg/mL and 1.95 μg/mL. The coefficients of determination (R^2^) for calibration curves were 0.998 for both compounds. The repeatability was measured by 3 replicate analyses of a mixture of β-myrcene and α-humulene at three different concentrations at the same day, whereas the intermediate precision on three consecutive days. The RSD values, which are calculated according to the equation: RSD% = (standard deviation of the peak area/mean) × 100, show that both repeatability (RSD% < 3) and intermediate precision (RSD% < 6) are acceptable (Table 2).

### 3.2. Gas Chromatography (GC-MS/MS)

The qualitative analyses of obtained hops oils were performed with a GC-MS instrument (Agilent 7890) equipped with mass spectrometry and NIST 2011 MS spectra library. As for the acquired results, Masshunter software (ver. C.01.03) was used. The following analyses parameters were used for *Humulus lupulus* essential oil samples: Capillary GC column DB-EUPAH (60 m × 250 μm; 0.25 μm) with helium as the carrier gas, and flow rate of 2 mL/min in a split injection mode (40:1). Oven temperature was set initially at 50 °C (held for 1 min) and increased to 260 °C at the rate of 6 °C/min (held for 6 min) and then finally reached 300 °C at the rate of 3 °C/min and held for 20 min. The system was operating in the EI mode (electron energy 70 eV) with the temperature of the source ion set to 230 °C and the scanning range from 40 to 650 amu. 

#### 3.2.1. Quantification

Concerning the quantitative analysis, internal standard calibration method was used with 4-androstene-3, and 17-dione as an internal standard. About 5 mg of the standard was weighed into a 5 mL volumetric flask. Then 5 mL of methanol/chloroform (9:1, *v*/*v*) mixture was added and 10 μL of such prepared standard was added to each oil sample in order to quantify β-myrcene and α-humulene. The standard curves were obtained from five concentration points: 200, 100, 50, 25, and 10 μg for β-myrcene and 50, 25, 10, 5, and 1 μg for α-humulene, adjusted to the real content of β-myrcene and α-humulene in the analyzed samples. The quantification was achieved in the MRM mode with different GC conditions as were used in the qualitative analyses. The quantitative method was shortened to 38 min with an oven temperature of 80 °C at the starting point, held for 1 min, and then ramped up to 300 °C with the rate of 10 °C/min and held for 15 min with nitrogen as a collision gas at the flow rate of 1.5 mL/min. The fragment ions for quantification and identification of each analyte were 136→121 (6) and 136→107 (6) for β-myrcene, 204→189 (10) and 204→161 (10) for α-humulene, as well as 286→148 (5), 124→109 (10) and 124→96 (10) for internal standard. The collision energies are provided in the brackets. 

#### 3.2.2. Partial Method Validation

Limits of detection (LOD) were 1.8 ng/mL and 2.5 ng/mL, whereas limits of quantification were 4.6 ng/mL and 8.0 ng/mL, respectively, for β-myrcene and α-humulene. The coefficients of determination (R^2^) for the calibration curves were 0.999 for both compounds. The mixture of β-myrcene and α-humulene at three different concentrations was injected on the same day as well as on three consecutive days to evaluate the repeatability and intermediate precision of the developed method. The RSD values indicate that both repeatability (RSD% < 3) and intermediate precision (RSD% < 6) are acceptable (Table 3).

## 4. Results and Discussion

### 4.1. Material for Extraction

During our previous studies different forms of hops were tested before the final material was chosen for the extraction. One of the materials was post-extraction residue (residue after hops extraction using scCO_2_) as a scattering element of the extract mixture with water. However, hop extract is characterized by its poor solubility in water [10,16]. The microwave hydrodistillation process was very rapid with the accompanying overheating of the raw material in an aqueous environment. During the optimization of the extraction process, attempts were made to replace the residues with an emulsifying substance. For this purpose, four different emulsifiers were used, including polysorbate 80 (E433), emulsifying biobase, soya lecithin, and cetyl alcohol. None of the used emulsifiers brought the expected results, meaning no adequate degree of mixing and homogenization was achieved. The local overheating of the material was also observed. Our studies were also performed on other forms of materials, such as raw hops and hops granules. In any case, no satisfactory effect and no amount of oils were obtained. It might have been caused by the low concentration of desirable compounds in the analyzed material. 

### 4.2. Extraction Results

The microwave-assisted hydrodistillation (MAHD) with various microwave power and hydrodistillation (HD) were performed on extracts of Marynka hops variety. In case of the Marynka extract, the yield obtained by MAHD with the microwave power of 295 W was similar to the yield obtained by means of the HD method (1.80% for MAHD vs. 1.90% for HD). The extraction time of MAHD was significantly shorter than that of the conventional HD (40 vs. 276 min). The maximum yield of the essential oil obtained from the Marynka extract was using MAHD with microwave power of 335 W (3.03%). Table 4 summarizes the results of MAHD and HD methods for the Marynka extract. 

**Figure 3:**
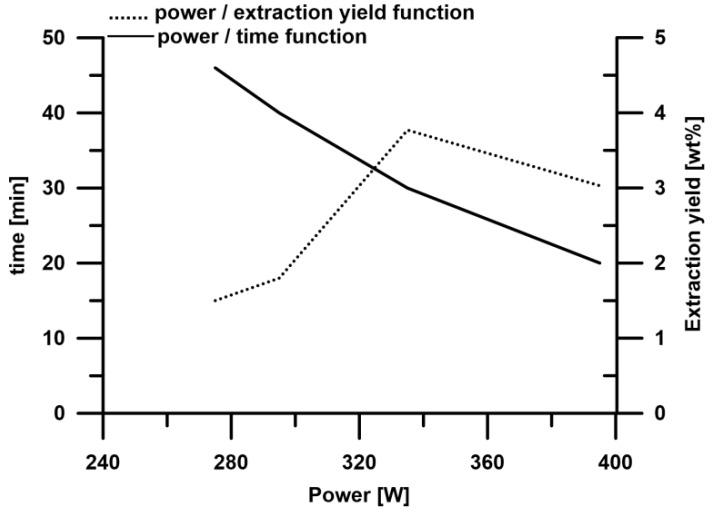
The influence of microwave power on the extraction time (––––) and the extraction yield (……….).

According to Routray and Orsat [22], the higher the extraction temperature, the better the yield can be obtained. However, when the optimal temperature is achieved, the extraction efficiency decreases. During the studies, a relationship between the extraction power and the extraction time was observed. With increasing microwave power, the extraction time was shortened. When the extraction was performed at 395 W (3.03%) the extraction yield was relatively lower in a comparison with the process at 335 W (3.77%). Figure 3 presents the influence of the extraction power on the extraction time and yield. 

**Figure 4:**
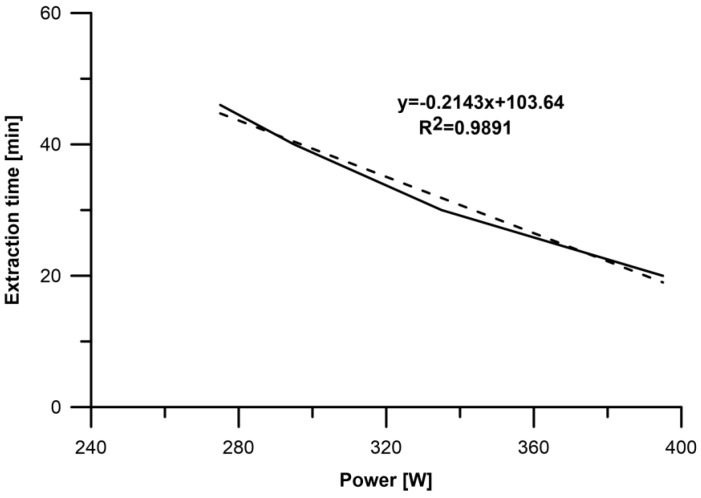
The function of the extraction time from the microwave power for the microwave-assisted hydrodistillation.

The function of the extraction time from the microwave power for the microwave-assisted hydrodistillation of the Marynka extract was determined. From the graph, the curve equation for the experimental data was read, from which the values for the extraction time with the applied microwave power values were determined. The obtained data showed that with the increase of the microwave power by 5 W, the extraction time is shorter by 1.07 min (Figure 4).

4.3. β-Myrcene and α-Humulezne Determination

Ligor et al. [23] obtained the essential oils from hops with a use of different techniques, such as supercritical fluid extraction, steam distillation, accelerated solvent extraction (ASE), and solid phase microextraction (SPME). The values for β-myrcene and α-humulene in obtained essential oils ranged from 15.7% to 21.1% and from 11.1% to 33.4%, respectively. Our study involves the use of microwave-assisted hydrodistillation, which is considered as a safe method [9]. The method resulted in obtaining the essential oil from *Humulus lupulus* scCO_2_ extract at optimized parameters of 30 min and 335 W, which corresponds to one third of the maximal working power of the system. 

The first application of the supercritical fluid chromatography with a photo-diode array detector to determine volatile compounds such as β-myrcene and α-humulene is provided in this study. This method was compared with a traditional method involving gas chromatography equipped with mass spectrometer (GC-MS/MS). As was suggested by Flament et al. [24], the use of scCO_2_ for the separation of volatile compounds is inappropriate. However, in this study the obtained results for β-myrcene and α-humulene were comparable. The SFC method appeared to be significantly shorter than GC-MS/MS. Moreover, the use of supercritical carbon dioxide is understandable here due to the non-polar nature of volatile compounds being a subject of analysis. According to SFC quantitative analysis of β-myrcene and α-humulene, confirmed by GC-MS/MS analysis, the obtained oils were enriched in β-myrcene in the amount of approx. 74.13%–89.32%, which was several times higher in a comparison with the values obtained by Ligor et al. [23]. In the case of α-humulene, the concentration ranged from 7.36% to 10.14% in the essential oils obtained by microwave-assisted hydrodistillation, whereas only 1.59% in the essential oil obtained by hydrodistillation. Table 5 summarizes the concentrations of β-myrcene and α-humulene in obtained essential oils analyzed by UPC^2^ and GC-MS/MS systems. 

## 5. Conclusions

Microwave-assisted hydrodistillation has been proven to offer advantages over hydrodistillation in determination of essential oils in *Humulus lupulus* scCO_2_ extract. A similar extraction yield of the essential oil from *Humulus lupulus* scCO_2_ extract is achieved in shorter extraction time (30 min for MAHD vs. 276 min for HD) and at a lower energy consumption (0.17 kWh/g of essential oil for MAHD vs. 1.89 kWh/g of essential oil for HD). Increasing the extraction time increases efficiency of the process as well as the amount of obtained compounds. Moreover, a significantly higher temperature and longer extraction time in the case of the hydrodistillation may have an influence on the compound’s thermal degradation. Both chromatographic methods, such as supercritical fluid chromatography (SFC) and gas chromatography-mass spectrometer (GC-MS/MS), are comparable with the advantage of the SFC method in terms of shortened analysis time. 

## Figures and Tables

**Table 1:** Preparation of samples for Marynka extract.

Sample Name	Charge Mass (g)	Extract Mass (g)	Water Mass (g)	Residue Mass (g) *
Marynka_275W	603.34	150.25	402.51	50.58
Marynka _295W	605.99	150.99	404.99	50.01
Marynka_335W	602.71	150.47	402.16	50.08
Marynka_395W	608.65	156.00	401.94	50.71
Marynka_Deryng	602.61	151.83	400.78	50.00

* Residue mass is the mass of the residue obtained after the extraction of *Humulus lupulus* with scCO_2_

**Table 2:** Calibration data as well as repeatability and intermediate precision for analyzing β-myrcene and α-humulene by UPC^2^ system.

Compound	Linearity Range (μg/mL)	R2	LOD (μg/mL)	LOQ (μg/mL)	Concentration (μg/mL)	RSD % (*n* = 3)	
						Repeatability	Intermediate Precision
β-myrcene	62.5–1000	0.998	0.89	2.23	75	2.16	3.87
					100	1.02	2.91
					200	0.72	2.36
α-humulene	31.125–500	0.998	0.78	1.95	75	2.00	3.12
					100	1.27	2.74
					200	0.50	2.03

**Table 3:** Calibration data as well as repeatability and intermediate precision for analyzing β-myrcene and α-humulene by GC-MS/MS system.

Compound	Linearity Range [μg/mL]	R2	LOD [ng/mL]	LOQ [ng/mL]	Concentration [μg/mL]	RSD % (*n* = 3)	
						Repeatability	Intermediate Precision
β-myrcene	10–200	0.999	1.8	4.6	50	2.36	3.25
					100	1.54	2.84
					200	0.98	1.99
α-humulene	1–50	0.999	2.5	8.0	50	2.47	3.17
					100	1.69	2.99
					200	1.12	2.39

**Table 4:** The results for Marynka extract essential oils microwave-assisted extraction.

Sample Name	Raw Material Mass (g)	Total Oil Amount (mg)	Yield (wt%)	Extraction Time (min)	Energy Consumption (kWh)
Marynka_275W	603.34	2248.14	1.50	46	0.215
Marynka_295W	605.99	2720.97	1.80	40	0.200
Marynka_335W	602.71	5676.44	3.77	30	0.170
Marynka_395W	608.65	4723.37	3.03	20	0.134
Marynka_Deryng	602.61	2807.31	1.90	276	1.897

**Table 5:** The results of quantitative analyses of β-myrcene and α-humulene in essential oils obtained by microwave-assisted hydrodistillation (MAHD) and hydrodistillation (HD).

Sample Name	β-Myrcene		α-Humulene	
	UPC2 (wt%)	GC-MS/MS (wt%)	UPC2 (wt%)	GC-MS/MS (wt%)
Marynka-275W	89.32	88.89	10.14	10.56
Marynka-295W	74.13	75.02	8.23	8.01
Marynka-335W	77.24	77.36	9.33	9.47
Marynka-395W	75.12	74.66	7.36	7.96
Marynka-Deryng	84.73	84.99	1.59	1.87
